# Elution of Radioactive Cesium from Tofu by Water Soaking

**DOI:** 10.14252/foodsafetyfscj.D-20-00011

**Published:** 2020-08-07

**Authors:** Mitsuru Yoshida, Hitomi Kaino, Saori Shidara, Kazuhiro Chiku, Mayumi Hachinohe, Shioka Hamamatsu

**Affiliations:** 1 Department of Food Science and Technology, Faculty of Applied Life Science, Nippon Veterinary and Life Science University, 1-7-1 Kyonancho, Musashino 180-8602, Japan; 2 Food Research Institute, National Agriculture and Food Research Organization, 2-1-12 Kannondai, Tsukuba 305-8642, Japan; 3 Headquaters, National Agriculture and Food Research Organization, 3-1-1 Kannondai, Tsukuba 305-8517, Japan

**Keywords:** ^137^Cs, bean curd, elution, soaking, tofu, radioactive cesium

## Abstract

Elution of cesium-137 (^137^Cs) from tofu into water was investigated to know the behavior of ^137^Cs during preservation and cooking. The food processing retention factor (*Fr*) reached 0.55 when tofu was soaked in water at a ratio of 1:2 *w*/*w* for 24 h at 4°C. Doubling the amount of water did not further significantly decrease *Fr*. When tofu was held in water at a ration of 1:2 w/w at a temperature of 80℃ for 50 min, *Fr* was 0.72. Increasing the amount of water to 10 times the tofu weight did not further reduce *Fr* significantly. Cesium-137 is mostly bound to tofu and does not freely diffuse into water. Tofu was then soaked in water at a ratio of 1:2 w/w at 4℃ for 24 h, placed in new water at a ratio of 1:2 w/w, and held at 80℃ for 50 min, resulting in an *Fr* 0.33. This value is close to an estimated *Fr* calculated by multiplying the *Fr* of 0.55 from soaking at 4°C by the *Fr* of 0.72 from the hot water treatment. The calculated *Fr* from soybeans sequentially processing into tofu, soaking tofu at 4°C for 24 h and in hot water at 80°C for 50 min was about 0.1, indicating 90% removal of ^137^Cs. Degree of decrease in ^137^Cs during preservation and cooking of tofu demonstrated in this study will be useful for exposure assessment of ^137^Cs through oral intake of contaminated soybeans after processing and cooking.

## 1. Introduction

Environmental pollution with radionuclides was caused by the tsunami that severely damaged the Fukushima Daiichi Nuclear Power Plant after the Great East Japan Earthquake on March 11, 2011. Contamination of agricultural products, mainly with radioactive cesium and iodide, became an issue in Fukushima Prefecture and the surrounding East Japan area just after the accident. The Japanese government set provisional standard values for radioactive cesium (^134/137^Cs) and iodine (^131^I) in drinking water and food on March 17, 2011. These values were determined assuming a 5 mSv upper limit for annual radiation dose from drinking water and food and using intake estimates for Japanese consumers. Contamination with ^131^I was resolved within a few months due to its short half-life of 8 days. Radioactive cesium isotopes, however, have longer half-lives, 2 and 30 years for ^134^Cs and ^137^Cs, respectively. Standard limits were newly set on April 1, 2014, decreasing the limit of annual radiation dose to 1 mSv and assuming that 50% of general foods and 100% of drinking water, milk and baby food are contaminated at limit values^1^^)^. The standard limits for radioactive Cs (^134^Cs + ^137^Cs) are 10 Bq/kg for drinking water, 50 Bq/kg for milk and baby food, and 100 Bq/kg for general food.

Cases where water or food exceeds standard limits are now rare due to natural radioactive decay, efforts for environmental remediation, and regulations based on monitoring of food by the government. Radioactive contamination of food is not currently a serious problem in Japan, but the accident of the nuclear facility illustrated the importance of intake estimation of radionuclides for setting standard limits for regulation. Standard limits for general food are applied to both raw and processed food, and processing raw materials generally decreases radioactive pollutants^2^^,^^3^^,^^4^^,^^5^^,^^6^^,^^7^^,^^8^^,^^9^^,^^10^^)^. More precise intake estimation of the pollutants should be possible by considering contaminant loss during processing and cooking.

Soybean is a major bean consumed in Japan. It is processed into bean curd, “tofu,” and “natto” through fermentation, in addition to oil, soy sauce, and “miso,” a fermented soybean paste. Home cooking also yields boiled soybean, “nimame.” Reduction of ^134^Cs and ^137^Cs in soybeans during processing into tofu, natto, and nimame has been reported^11^^)^. For soybean processing into tofu, 40% of radioactive Cs in soybeans, which distributed into water by washing and soaking beans and tofu refuse, is removed. Elution of Cs into water used for washing and soaking reflects the hydrophilicity of the contaminant. Tofu is a popular soybean-based food in Japan, which is commonly preserved in water and often cooked in water at stores, restaurants, and home. To understand the behavior of radioactive Cs in tofu during preservation and cooking, we investigated elution of ^137^Cs from tofu into water when kept under different conditions.

## 2. Materials and Methods

### 2.1 Soybean Samples

The Japanese soybean cultivar Tachinagaha used in this study was sown in June 2011 in a test field in Fukushima Prefecture and harvested in November 2011. Soybeans were stored at 4°C until use. The concentration of ^137^Cs in dried soybeans was 156 Bq/kg.

### 2.2 Preparation of Tofu and Water Soaking

Dried soybeans (150 g) were soaked in 900 mL of ion-exchange purified water at 4°C for 24 h. The water was removed, and water was newly added to the soaked soybeans to bring the total weight to 1000 g. The mixture was homogenized with a food processor (DLC-1J Mini-Prep Processor; Conair Japan G.K., Tokyo, Japan). The homogenate was heated on an induction heating cooking heater (KZ-PH33; Panasonic Co., Osaka, Japan) after addition of 10 mL canola oil (J-Oil Mills Inc., Tokyo, Japan) as an antifoaming agent. After the homogenate temperature reached 85°C, heat was maintained for 15 min. The heated homogenate was filtered with a nylon meshed sheet to obtain soymilk. Soymilk was cooled in an ice bath, and 0.5% by weight of d-glucono-1,5-lactone (FUJIFILM Wako Pure Chemical Co., Osaka, Japan) was added. Soymilk was then heated in a boiling water bath for about 1 h until it solidified into tofu. The tofu was cut into 4 × 6 × 3.5 cm (about 100 g) pieces and soaked in ion-exchange purified water under prescribed conditions. Soaking experiments were run in triplicate using different batch of tofu.

### 2.3 Radioactivity Measurement

Tofu was packed into a 100-mL U-8 container (PP-U8; Sekiya Rika, Co. Ltd., Tokyo, Japan), and soaking water was decanted into a 0.7-liter Marinelli container (SMAX-Y07, Sekiya Rika Co. Ltd.). Concentrations of ^137^Cs were measured by γ-ray spectrometry with germanium semiconductor detectors (GC2020, GC2520, GC4020; Canberra, Meriden, CT, USA) coupled to a multichannel analyzer (DAS1000; Canberra). Cesium-137 concentrations were calculated using Spectrum Explorer 1.71 software (Canberra) integrated with the germanium semiconductor detector. The γ-ray peak used in ^137^Cs measurements was 661.6 keV. Counting times were set so that the counting error was <10% of the total count, and ^137^Cs concentration is reported as the γ-activity at 0:00:00 on February 1, 2019 in Japanese Standard Time.

The processing factor (*Pf*) was calculated as follows: *Pf* = *A*/*B*, where *A* is the ^137^Cs concentration after processing (Bq/kg [fresh wt]), and *B* is the ^137^Cs concentration before processing (Bq/kg [fresh wt]). The food processing retention factor (*Fr*), was calculated as follows: *Fr* = *C*/*D*, where *C* is the ^137^Cs content after processing (Bq), and *D* is the ^137^Cs content before processing (Bq) based on sample mass. The data from the same batch of tofu were regarded as a set, and *Pf* and *Fr* were calculated using data within a set. Significant differences (*p* < 0.05) in *Fr* between two treatments were assessed with a paired *t*-test as the values from the same batch of tofu were regarded as a pair.

## 3. Results and Discussion

The average concentration of ^137^Cs in prepared tofu was 16 Bq/kg. The *Pf* of tofu prepared from dried soybeans was 0.10, which is similar to values reported by Hachinohe et al.^11^^)^. The *Fr* for tofu prepared in this study was 0.28, which is about half of that previously reported (0.64)^11^^)^. This difference may be due to differences in conditions of soaking, homogenization, and separation of soymilk. Recovery of ^137^Cs in all soaking experiments calculated using *Fr* of tofu and soaking water was 0.83–1.28.

**Fig. 1.**
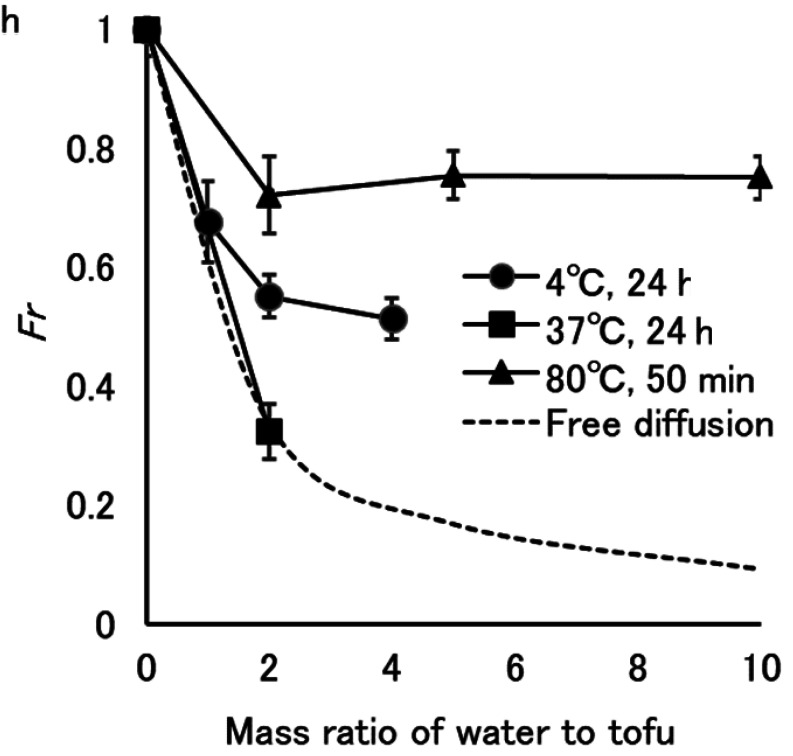
Effect of water mass on *Fr* of tofu. Dotted line indicates *Fr* when ^137^Cs concentration in tofu becomes equal to that in water by free diffusion.

A loaf of tofu was soaked in water at 4°C for 24 h, simulating storage at home. After soaking in water with twice the weight of tofu, *Fr* reached 0.55 (Fig. 1, closed circle). Increasing the amount of water up to four times the weight of tofu did not further decrease *Fr* significantly (*p* ≥ 0.05). The *Fr* should be 0.33 and 0.20 when ^137^Cs concentration in tofu becomes equal to that in water which is twice and four times the weight of tofu, respectively (Fig. 1, dotted line). Thus, more than half the ^137^Cs is considered to be trapped in tofu.

When the temperature was raised to 37°C, *Fr* was reduced to 0.33 in water twice the weight of tofu for 24 h (Fig. 1, closed square). The residual ratio of ^137^Cs in tofu at 37°C is lower than that at 4°C, but a temperature of 37°C is not hygienic for the preservation of tofu.

Tofu is often served in hot seaweed-based broth as “yudofu” in Japan. We investigated elution of ^137^Cs into hot water over shorter periods of time. Tofu was added to twice its weight of boiling water, and the temperature was then maintained at 80°C for 50 min. The core temperature of the tofu reached 80°C in 20 min and was maintained for 30 min in this condition. This treatment decreased *Fr* to only 0.72 from that of tofu before added to boiling water. Increasing the amount of water up to 10 times the weight of tofu did not further significantly (*p* ≥ 0.05) reduce *Fr* (Fig. 1, closed triangle). Cesium-137 did not freely diffuse from tofu into hot water over periods of time of less than 50 min. How ^137^Cs is trapped in tofu requires investigation in further research.

We further investigated the combined effects of soaking and cooking in water on release of ^137^Cs from tofu. Tofu was soaked in twice its weight of water at 4°C for 24 h, then shifted into twice its weight of boiling water and maintained at 80°C for 50 min resulting in an overall *Fr* of 0.33. This value is close to that estimated by multiplying the *Fr* of 0.55 from refrigeration by the *Fr* of 0.72 from the hot water treatment. The calculated *Fr* from soybeans through processing into tofu, soaking the tofu at 4°C for 24 h, and then cooking in water at 80°C for 50 min was about 0.1, indicating 90% removal of ^137^Cs.

Decrease in radioactive Cs by processing soybeans into tofu and during preservation and cooking of tofu in water is demonstrated in this study. This information will be useful for exposure assessment of ^137^Cs by oral intake of contaminated soybeans after processing and cooking.
